# Surface Proteins of *Lactococcus lactis:* Bacterial Resources for Muco-adhesion in the Gastrointestinal Tract

**DOI:** 10.3389/fmicb.2017.02247

**Published:** 2017-11-23

**Authors:** Muriel Mercier-Bonin, Marie-Pierre Chapot-Chartier

**Affiliations:** ^1^Toxalim (Research Centre in Food Toxicology), Université de Toulouse, INRA, ENVT, INP-Purpan, UPS, Toulouse, France; ^2^Micalis Institute, INRA, AgroParisTech, Université Paris-Saclay, Jouy-en-Josas, France

**Keywords:** lactic acid bacteria, *Lactococcus lactis*, mucus, adhesion, surface proteins, pili, mucus-binding protein

## Abstract

Food and probiotic bacteria, in particular lactic acid bacteria, are ingested in large amounts by humans and are part of the transient microbiota which is increasingly considered to be able to impact the resident microbiota and thus possibly the host health. The lactic acid bacterium *Lactococcus lactis* is extensively used in starter cultures to produce dairy fermented food. Also because of a generally recognized as safe status, *L. lactis* has been considered as a possible vehicle to deliver *in vivo* therapeutic molecules with anti-inflammatory properties in the gastrointestinal tract. One of the key factors that may favor health effects of beneficial bacteria to the host is their capacity to colonize transiently the gut, notably through close interactions with mucus, which covers and protects the intestinal epithelium. Several *L. lactis* strains have been shown to exhibit mucus-binding properties and bacterial surface proteins have been identified as key determinants of such capacity. In this review, we describe the different types of surface proteins found in *L. lactis*, with a special focus on mucus-binding proteins and pili. We also review the different approaches used to investigate the adhesion of *L. lactis* to mucus, and particularly to mucins, one of its major components, and we present how these approaches allowed revealing the role of surface proteins in muco-adhesion.

## Introduction

Lactic acid bacteria (LAB), used as starters in food fermentations and as probiotics, are regularly ingested in large amount by humans. After their ingestion, these bacteria temporarily co-exist with the complex resident gut microbiota. Emerging evidence suggests that this transient microbiota has an impact on composition and metabolism of the gut microbiota and thus possibly on host health (Veiga et al., 2014; Derrien and van Hylckama Vlieg, 2015). *Lactococcus lactis* is one of the most widely used LAB in starter cultures for manufacturing dairy fermented products. Besides, several natural isolates have been described with beneficial health properties and recombinant *L. lactis* has been proposed as a delivery vehicle for therapeutic molecules in the gastrointestinal tract (GIT) (Carvalho et al., 2017). Transient colonization of the gut by the ingested bacteria, notably through adhesion to mucus that covers and protects the intestinal epithelium, is expected to favor their beneficial effect on the host. The ability of commensal or pathogenic bacteria to adhere to intestinal mucus glycoconjugates was previously attributed to specific proteins present at the bacterial surface (Kline et al., 2009; Hill, 2012; Juge, 2012; Moonens and Remaut, 2017). Although *L. lactis* is not a natural inhabitant of the mammalian GIT, proteins able to bind mucins have also been described. This mini-review gives an overview of the beneficial effects reported for *L. lactis*, the mucus composition and function, the different surface proteins discovered in *L. lactis* and involved in muco-adhesion and the different complementary approaches and tools used to uncover their role.

## Beneficial Effects of Lactococci in the Git

The main beneficial effects reported for natural or recombinant *L. lactis* strains concern their anti-inflammatory potential, making them as possible therapeutic tools in human intestinal bowel diseases. First, several natural *L. lactis* isolates were shown to possess anti-inflammatory properties in cellular models as well as *in vivo* in mouse models of intestinal colitis (Nishitani et al., 2009; Luerce et al., 2014; Ballal et al., 2015). Besides, recombinant *L. lactis* expressing anti-inflammatory molecules (cytokine IL10, anti-oxidant enzymes, or protease inhibitor elafin) efficiently reduce intestinal inflammation symptoms and restore colon homeostasis in mice (Bermudez-Humaran et al., 2013). Alleviation of food allergic manifestations in sensitized mice was also described for *L. lactis* NCC2287 (Zuercher et al., 2012). Interestingly, a recent study reported that *L. lactis* present in a fermented milk product was able to modulate the gut microbiota in permissive subjects (rats or humans), and this property was correlated with an increased persistence in the GIT (Zhang et al., 2016).

## Mucus and Mucins in the Git

The mammalian GIT is covered by mucus, a viscoelastic gel that lines and protects the intestinal epithelium, separating it from the lumen content. Mucus functions as a dynamic barrier that is permeable to gasses, water and nutrients, but impermeable to most microorganisms. This substance was long considered to act as a “simple” physical barrier, but is now known to exert other key functions essential for maintaining intestinal homeostasis (Juge, 2012; Ouwerkerk et al., 2013). Mucus covers the intestinal epithelium to a different extent along the GIT. In lower GIT, the small intestine has only a single layer whereas the colon displays a two-layered mucus (Ermund et al., 2013). The inner layer is depicted as essentially sterile in contrast with the outer layer which is highly colonized (Donaldson et al., 2016). The main constituents of mucus are mucins, which are produced, stored and released by goblet cells. Mucins are large glycoproteins in which the glycans constitute more than 80% of the molecular mass. The O-linked glycan chains contain 1–20 sugar residues most commonly attached to the protein backbone through serine or threonine with *N*-acetylgalactosamine (GalNAc). The chain is elongated with core structures and two potential backbone regions containing *N*-acetylglucosamine (GlcNAc) and galactose (Gal). Terminal sugars are usually fucose, Gal, GalNAc, or sialic acid residues and both Gal and GlcNAc residues may be sulfated, thus offering a high degree of diversification (Linden et al., 2008). MUC2 is the secreted gel-forming mucin present in the intestine (Johansson et al., 2011). In humans, MUC2 is coated with more than 100 different O-linked glycan chains (Larsson et al., 2009), which notably differ from those in rodents (Thomsson et al., 2012). Mucin oligosaccharides can serve both as binding sites and energy sources for GIT microbes and the difference in the glycan “preferences” of bacteria was suggested to explain host specificity in terms of microbiota (Donaldson et al., 2016).

## *L. lactis* Surface Proteins Involved in Muco-Adhesion

The cell wall of Gram positive bacteria is made of a thick peptidoglycan sacculus decorated with other glycopolymers (polysaccharides and teichoic acids) as well as proteins (Chapot-Chartier and Kulakauskas, 2014). The cell wall proteins are usually classified based on their mode of attachment to the cell envelope: (i) covalent attachment to peptidoglycan through an LPxTG motif and a sortase A-mediated reaction; (ii) non-covalent binding to cell wall glycopolymers through specific domains (e.g., LysM or SH3 domains); (iii) lipid anchoring in the membrane (lipoprotein); (iv) one or several transmembrane domains.

Among the cell wall proteins, only those protruding at the external surface, that are surface-exposed proteins constituting the surfome (Olaya-Abril et al., 2014), are likely involved in bacterial adhesion to abiotic and biotic surfaces. These proteins can be predicted *in silico* by specific flow scheme such as Surf G+ (Barinov et al., 2009). At the experimental level, they can be specifically targeted by dedicated proteomic methods such as (i) the “shaving” procedure consisting in proteolytic digestion of surface-exposed proteins on live bacteria and LC-MS/MS identification of released peptides (Olaya-Abril et al., 2014) or (ii) selective labeling with a fluorescent dye followed by 2D gel analysis (Le Marechal et al., 2015).

Although *L. lactis* is not a typical inhabitant of the mammalian GIT, several surfaces proteins have been previously identified with the ability to bind to mucus/mucins. Most of them belong to the LPxTG-protein family and are encoded by plasmids. Of note, plasmids significantly contribute to the genetic diversity encountered in the *L. lactis* species (Ainsworth et al., 2014), related to its adaptation to different niches (food, plant, or animal) (Kelly et al., 2010; Garrigues et al., 2013; Kelleher et al., 2017; Laroute et al., 2017). Thus, whereas the laboratory strains, obtained from dairy strains by plasmid and prophage curing, are considered to have a restricted surface proteome (Habimana et al., 2007), the *L. lactis* surface pan-proteome is probably much richer and more diverse, constituting a potential reservoir of muco-adhesive factors. The proteins described below are likely contributing to muco-adhesion, either through non-specific hydrophobic interactions with mucus components (aggregation factor AggL, protease PrtP) or specific binding to mucin glycans [mucus-binding proteins (MUBs), pilins containing “lectin” domains]. In this latter case, it can be speculated that, in the original ecological niche of *L. lactis*, i.e., plant, these proteins might play a role in binding sugar motifs at the surface of vegetal cells, and these motifs could be shared by mucin sugars (Meyrand et al., 2013).

### Mucus-Binding Proteins

Mucus-binding proteins are bacterial surface adhesins with typical signal peptide and C-terminal LPxTG motif. They contain (multiple) Mub domains (around 200 residues in length) and/or MucBP domains (Pfam PF06458, around 50 residues), which have been shown to bind mucins (Juge, 2012). According to structural studies, the Mub domains consist of two modules B1 and B2 with B2 being a MucBP domain (MacKenzie et al., 2009; Etzold et al., 2014). Contrary to the MucBP domain also present in pathogens (Popowska et al., 2017), the Mub domain is predominantly found in LAB and is highly abundant in lactobacilli of the gut microbiota (Boekhorst et al., 2006). The best characterized MUB protein from *Lactobacillus reuteri* endowed with 14 Mub domains (Roos and Jonsson, 2002) binds mucus via multiple interactions involving terminal sialylated mucin glycans (Etzold and Juge, 2014; Gunning et al., 2016). In *L. lactis* plasmid free laboratory strains IL1403 and MG1363, only one MUB protein with four Mub domains and devoid of signal sequence, is encoded in the chromosome sequence (Boekhorst et al., 2006). However, these strains do not exhibit muco-adhesive properties (Radziwill-Bienkowska et al., 2017), suggesting that this MUB protein is not expressed or not functional. In contrast, three other MUB proteins found in dairy or vegetal strains were shown to contribute to bacterial adhesion to mucins as tested by different methods and tools (**Table 1**). These three proteins have different structures (**Table 1**), with three MucBP domains in MbpL protein from dairy *L. lactis* BGKP1 (Kojic et al., 2011), two Mub domains in Muc protein from vegetal *L. lactis* TIL448 (Meyrand et al., 2013) and one MucBP domain in AJ89_07570 protein from dairy *L. lactis* IBB477 (Radziwill-Bienkowska et al., 2016).

**Table 1:** Surface proteins identified as responsible for muco-adhesive properties in different natural *L. lactis* strains and corresponding adhesion tests used to probe adhesion to mucus or mucins.

Protein (length)	Characteristics	Adhesion test	Reference
***L. lactis* subsp. *lactis* BGKP1 (dairy)**			
**MbpL**	Signal peptide	*In vitro*: pig gastric mucin in microtiter plate	Kojic et al., 2011
(998 a.a.)	LPxTG motif	*In vitro*: HT29-MTX intestinal epithelial cell line (muco-secreting)	Lukic et al., 2012
(plasmid)	Three MucBP domains		
			
**AggL**	Signal peptide	*In vitro*: pig gastric mucin in microtiter plate	Kojic et al., 2011
(1767 a.a.)	LPxTG motif	*Ex vivo* colonic mucus	Lukic et al., 2012
(plasmid)	Four Collagen_bind domains (PF05737)	*In vivo* in rat	
	Six CnaB domains (PF05738)		
			
***L. lactis* subsp. *lactis* TIL448 (NCDO2110) (plant)**			
**Muc**	Signal peptide	*In vitro*: pig gastric mucin by AFM	Meyrand et al., 2013
(1130 a.a.)	LPxTG motif	*In vitro*: pig gastric mucin in shear stress flow chamber	Le et al., 2013
(plasmid)	Two Mub domains		
			
**Pili**	Signal peptide	*In vitro*: pig gastric mucin by AFM	Meyrand et al., 2013
(Tip pilin)	LPxTG motif	*In vitro*: pig gastric mucin in shear stress flow chamber	Le et al., 2013
(817 a.a.) (plasmid)	*Lectin_legB* domain		
			
***L. lactis* subsp. *cremoris* IBB477 (dairy)**			
**AJ89_07570**	Signal peptide	*In vitro*: pig gastric mucin in microtiter plate	Radziwill-Bienkowska et al., 2016
(956 a.a.)	One DUF285 domain		
(chromosome)	One MucBP domain		
	Four Big_3 domains		
			
**PrtP**	Signal peptide	*In vitro*: pig gastric mucin in microtiter plate	Radziwill-Bienkowska et al., 2017
(1960 a.a.)	LPxTG motif	*In vitro*: HT29-MTX intestinal epithelial cell line (muco-secreting)	
(plasmid)		*In vivo* in mice	

**FIGURE 1:**
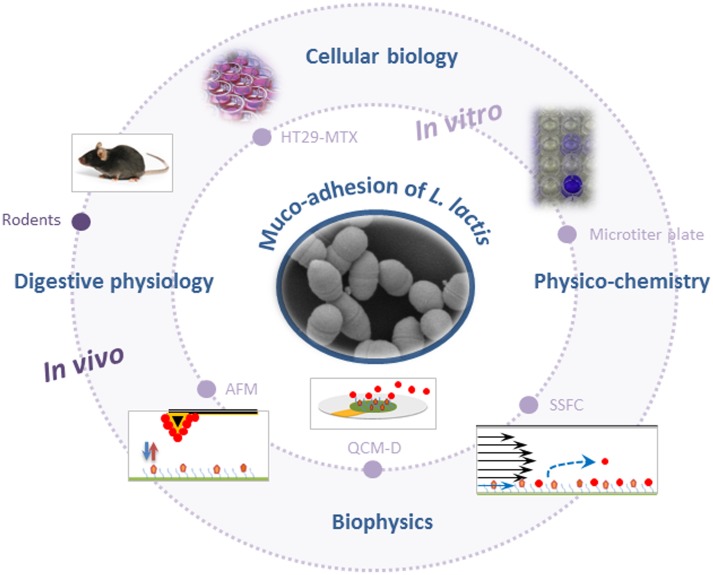
Schematic representation of methods and tools used to probe muco-adhesion of *Lactococcus lactis* (AFM, atomic force microscopy; QCM-D, quartz crystal microbalance with dissipation monitoring; SSFC, shear stress flow chamber). For the sake of clarity, schemes are not to scale.

*The different adhesion tests are schematically presented in **Figure 1**.*

### Pili

Pili are elongated protein structures of 1–10 nm in diameter and a few μm in length, protruding outside bacterial cells. They were long considered as characteristic of pathogens (Danne and Dramsi, 2012) but have been later discovered in LAB, in probiotic *Lactobacillus rhamnosus* GG, where they bind human mucins and could explain the strain persistence in human GIT (Kankainen et al., 2009). Pili were also described in *L. lactis*. Until now, three different gene clusters specifying sortase-dependent heterotrimeric pili have been identified in several *L. lactis* strains, one chromosomal present in all *L. lactis* strains (Oxaran et al., 2012) and two plasmid-encoded others in plant (Meyrand et al., 2013) and dairy (Tarazanova et al., 2016) strains respectively. These gene clusters encode three pilins, the major pilin which is polymerized by the sortase C to form the pilus backbone, a minor anchoring pilin and a tip pilin usually endowed with adhesive properties, as well as sortase C (Mandlik et al., 2008; Hendrickx et al., 2011). After synthesis, pili are covalently anchored to peptidoglycan by sortase A (Dieye et al., 2010). Noteworthy, the three identified *L. lactis* pili gene clusters exhibit limited sequence identity and have different syntheny. The chromosomal pilus gene cluster studied in the laboratory strain IL1403, as well as the plasmid one in *L. lactis* NCDO712 dairy strain, are not expressed in standard conditions but overexpression allowed pili synthesis (Oxaran et al., 2012; Tarazanova et al., 2016). In contrast, in the plant isolate *L. lactis* TIL448, the synthesis of pili at the bacterial surface was revealed without overexpression, by a proteomic analysis with the “shaving” approach and the presence of pili was visualized by electron microscopy and atomic force microscopy (AFM). These pili were further shown to contribute, together with the protein Muc, to mucin specific binding (**Table 1**). Interestingly, the tip pilin is a large protein with an LPxTG motif and containing a lectin domain (PF00139, *Lectin_legB* domain) that could recognized mucin glycan (Meyrand et al., 2013). Pili were also visualized by electron microcopy in four other *L. lactis* plant or clinical natural isolates but the genes involved in their synthesis were not identified (Oxaran et al., 2012).

### Aggregation Factor AggL

A plasmid-encoded aggregation factor, AggL, was characterized in *L. lactis* BGKP1. It is a cell-wall anchored protein with a LPxTG motif and seven collagen-binding protein B domains (CnaB domain) and it is responsible for cell surface hydrophobicity and bacterial aggregation phenotype (Kojic et al., 2011). It was shown to contribute to mucus adhesion (**Table 1**), probably as a result of non-specific hydrophobic interactions with the hydrophobic mucosal surface (Lukic et al., 2012).

### Cell Wall Protease PrtP

The cell-wall anchored proteinase, PrtP, has a crucial role in milk casein degradation required for efficient growth of *L. lactis* in milk (Siezen, 1999). The presence of PrtP at the surface of *L. lactis* was shown to modify the cell surface physico-chemical properties, leading to a greater hydrophobicity and increased adhesion to abiotic surfaces (Habimana et al., 2007). In *L. lactis* IBB477, the cell surface PrtP was shown to contribute to mucin adhesion (Radziwill-Bienkowska et al., 2017) (**Table 1**).

## Tools to Decipher the Role of *L. lactis* Surface Proteins in Muco-Adhesion

The different tools and methods used until now to probe adhesion of *L. lactis* to mucus/mucins are summarized in **Figure 1**.

### *In Vitro* Models

*In vitro* models that are most commonly used for evaluation of *L. lactis* adhesive properties, involve static microtiter plate assays using immobilized commercially available mucin (pig gastric mucin, PGM) and/or gut-related epithelial cell lines (**Figure 1**). Adherent bacterial cells are quantified using various approaches such as culturing/microscopical enumeration (Kimoto et al., 1999; Meyrand et al., 2013), radiolabeling (Rintahaka et al., 2014), crystal violet staining (Lukic et al., 2012; Radziwill-Bienkowska et al., 2016), or FISH (Radziwill-Bienkowska et al., 2017). The two most commonly used intestinal epithelial cell lines to study LAB adhesion are Caco-2 and HT29, which are originally derived from a human colorectal adenocarcinoma. Their major disadvantage is the lack of substantial mucus production. However, a mucus-secreting subpopulation of goblet cells from HT29 cell population (HT29-MTX) has been obtained after growth adaptation of HT29 cells to anticancer drug methotrexate (MTX) (Lesuffleur et al., 1990) and this cell line is increasingly used to investigate the muco-adhesive properties of *L. lactis* (Lukic et al., 2012; Radziwill-Bienkowska et al., 2017).

### Biophysics-Based Concepts and Tools

Biophysics-based tools have provided new *in vitro* insights into the interaction mechanisms between *L. lactis* and mucins (**Figure 1**). Interaction forces were quantified at nanoscale by AFM force spectroscopy using a *L. lactis-*functionalized tip (“lacto-probe”) and PGM-coated surface (Dague et al., 2010). Both non-specific and specific forces (ligand/receptor bonding) were at play in *L. lactis* adhesion to PGM. For *L. lactis* IBB477, the percentage of specific adhesive events was high (20%), in comparison with its low-adhesive counterpart *L. lactis* MG1820 (about 5%) (Le et al., 2011). Furthermore, mucin oligosaccharides were essential in interactions between *L. lactis* and PGM, as shown with blocking assays. For the first time with living cell probes and mucin, specific interactions were analyzed through kinetic constants: (i) the kinetic dissociation constant K_off_ was determined with increasing the tip loading rate, which led to a higher adhesion force. The K_off_ value (0.46 s^-1^) was consistent with values corresponding to sugar/protein interactions (Le et al., 2011); (ii) the kinetic association constant K_on_ (3.3 × 10^2^ M^-1^ s^-1^) was deduced from enhancing the tip/sample contact time. Furthermore, the high adhesion of IBB477 *vs.* MG1820 to PGM was confirmed at the multicellular level and under flow field conditions, using real-time quartz crystal microbalance with dissipation monitoring (QCM-D) (Le et al., 2012) and shear stress flow chamber (Radziwill-Bienkowska et al., 2016). Later studies revealed the combined contribution of chromosomal and plasmid-encoded cell-wall proteins (MucBP-Big_3 containing protein and proteinase PrtP respectively) in the muco-adhesive properties of IBB477 (Radziwill-Bienkowska et al., 2016, 2017).

A further work on the plant isolate *L. lactis* TIL448 enabled to elucidate the *L. lactis* muco-adhesive phenotype, based on the respective contribution of pili and MUB protein (Le et al., 2013). With AFM force spectroscopy, a high proportion of specific adhesive events to PGM was detected (60%), consistent with the weak shear-flow induced detachment of bacterial cells from the PGM coating. Rupture events were observed in AFM at short (100–200 nm) and long distances (up to 600–800 nm). With AFM force spectroscopy on pili and MUB protein defective mutants, the equivalent role exerted by these two cell surface determinants was established. However, under shear flow, a more critical contribution of MUB protein than pili was found. The importance of pili was further dissected in relation with their nanomechanical properties as probed with optical tweezer (Castelain et al., 2016a,b). AFM blocking assays also revealed that mucin neutral oligosaccharides were involved in adhesion of *L. lactis* TIL448 to PGM (Le et al., 2013).

### *In Vivo* Animal Models

A relatively low number of *in vivo*/*ex vivo* studies (i.e., biopsy samples) have been conducted to confirm the muco-adhesive phenotype of *L. lactis* in “real” environmental conditions (**Figure 1**). Application of such methods seems to be the next step, as shown for lactobacilli (Da Silva et al., 2015; Walsham et al., 2016). Nevertheless, first experimental evidence has been provided for lactococci. In mice, the GFP-labeled *L. lactis* WH-C1 strain was found to adhere to the gut mucosa (Wang et al., 2011). In another study in rats, in *ex vivo* and *in vivo* experiments, AggL protein was found to confer adhesive properties to *L. lactis* BGKP1 to colonic tissue through non-specific hydrophobic interactions. In contrast, for this particular strain, the MbpL protein did not contribute to bacterial adhesion to colonic tissues but was rather involved in gastric mucin binding (Lukic et al., 2012). More recently, it was shown that the cell-wall proteinase PrtP, albeit contributing to *in vitro* muco-adhesion of *L. lactis* IBB477, probably through non-specific interactions, could not confer a selective advantage to this strain in the gut of conventional C57BL/6 mice (Radziwill-Bienkowska et al., 2017).

## Conclusion

Certain *L. lactis* strains synthesize surface proteins with muco-adhesive properties. Although *L. lactis* is not a natural inhabitant of the mammalian GIT, the main classes of bacterial adhesins, i.e., MUB proteins and pili, allowing mucin glycan recognition, are encoded in the pangenome of the *L. lactis* species, which includes numerous plasmids. In commensal lactobacilli, MUB proteins and pili promote host mucosae colonization, while in *L. lactis* in its original ecological niche these proteins might rather play a role in plant tissue colonization. Nevertheless, the lactococcal pangenome appears as a reservoir of novel functions for beneficial gut-targeted activity. Indeed, muco-adhesive *L. lactis* strains will probably display an augmented fitness in the host GIT, favoring transient colonization, and thus potentially promoting health benefits. Until now, *L. lactis* muco-adhesion has been mainly studied *in vitro* with a wide range of tools and approaches on PGM and HT29-MTX cells, that should be completed in the future with *in vitro* studies on human intestinal mucins and *in vivo* studies.

## Author Contributions

All authors listed have made a substantial, direct and intellectual contribution to the work, and approved it for publication.

## Conflict of Interest Statement

The authors declare that the research was conducted in the absence of any commercial or financial relationships that could be construed as a potential conflict of interest.
